# 

**Published:** 1824-01

**Authors:** 


### THE LONDON MEDICAL AND PHYSICAL JOURNAL.

CONTAINING

ORIGINAL CORRESPONDENCE OF EMINENT PRACTITIONERS,

AND

CRITICAL ANALYSIS OF NEW WORKS

RELATING TO MEDICINE, SURGERY, MIDWIFERY, CHEMISTRY, PHARMACY BOTANY, AND NATURAL HISTORY.

---

EDITED BY

RODERICK MACLEOD, M.D.

LICENTIATE OF THE COLLEGE OF PHYSICIANS OF LONDON; MEMBER OF THE MEDICO-CHIRURGICAL SOCIETY OF LONDON, AND OF THE ROYAL MEDICAL SOCIETY OF EDINBURGH; PHYSICIAN TO THE WESTMINSTER GENERAL DISPENSARY; TO THE INFIRMARY FOR CHILDREN, AND TO THE SCOTTISH HOSPITAL; LECTURER ON THE PRACTICE OF PHYSIC AND MATERIA MEDICA, AT THE MEDICAL THEATRE, WINDMILL-STREET:

AND

JOHN BACOT, Esq.

MEMBER OF THE ROYAL COLLEGE OF SURGEONS; SURGEON TO THE ST. GEORGE'S AND ST. JAMES'S DISPENSARY; AND LATELY SURGEON TO HIS MAJESTY'S GRENADIER REGIMENT OF FOOT GUARDS.

---

VOL. LI.

FROM JANUARY TO JUNE, 1824.

Et quoniam variant morbi, variabimus artes;

Mille mali species, mille salutis erunt.

Surgeon Gen'l's Office LIBRARY 45584

*LONDON:*

PRINTED FOR THE PROPRIETORS,

*By J. and C. Adlard, Bartholomew Close;*

PUBLISHED BY J. SOUTER, 73, ST. PAUL'S CHURCH-YARD; AND MAY BE HAD OF ALL BOOKSELLERS.

W1
LO 31

### INDEX

OF

THE FIFTY-FIRST VOLUME

OF THE

### London Medical and Physical Journal.

PAGE

ANEURISM of the Aorta, case of . 341
Axillary Aneurism, account of . 147
Actæa Racemosa, in Phthisis Pulmonalis . . . 256
Artery, diseased, case of . . 148
Anus, imperforate, case of . 258
Apoplexy, Meningeal, case of . 364
Ascites, case of . . . 144
Alkali, new, in Jalap . . 346
Anatomy of the Eye, on the . 3
——— of the Cassowary . 165
Animals, on the reproduction of 22
Aneurism, remarks on . . 30
Acupuncturation, reference to cases of . . . . . 39
Apparatus for compressing Aneurism . . . . . 46
Anatomy of the Ear, on the minute . . . . . 16
Aquatic Salamander, reproduction of the members of . . 22
Arteries, on the temporary ligature of . . . . . 46

Brain, peculiarities of, in Fishes . 2
Books, Catalogues of, 85, 173, 261, 348, 437, 525
Brain, imperfect development of . 25
Biliary Calculi, cases of . . 139
Burns, cases of . . . 47
Bladder, on the injection of the . 145
Brain, experiments on the . 252
Bronchocele, case of . . 141
Brain, on the pathology of . 400
——— softening of the . . 28
——— remarks on . . 1

Cancer, on diseases resembling . 64
Calculi and Stricture, remarks on 47
Colchicum, remarks on . . 181
Correspondents, Notices to, 86, 262, 438, 526
Cellular texture, inflammation of . 503
Compression and Percussion, in Rheumatism . . . 446
Cases of Burns . . . 47

PAGE

Case of reversed position of the Viscera . . . . 474
——— diseased Artery . . 148
——— imperforate Anus . 258
——— male Twins united . 292
——— Bronchocele . . 141
——— Convulsion from Worms . 434
——— Malformation of Infant . 175
——— Caries of the Ribs . . 114
——— Stricture cured by incision 142
——— Infantile Disease . . 498
——— complicated Labour . 143
——— internal Hemorrhage . 289
——— Hydrocephalus Chronicus . 102
——— Biliary Calculi . . 139
——— Cephalalgia . . 177
——— Fistulous Opening in the Trachea . . . . 254
——— Tumor removed from the Conjunctiva . . . 344
——— Ulceration of the Palate . 389
——— Strangulated Exomphalos 149
——— Meningeal Apoplexy . 364
——— Ascites . . . 144
——— Perforation of Stomach . 433
Cases of Varioloid Disease . 299
——— Phlegmasia Dolens . 129
——— Neuralgia . . 271
Caries of the Ribs . . 114
Cataract, observations on . 423
Calculi, Biliary, on . . 139
Cephalalgia, case of . . 177
Conjunctiva, Tumor on the . 344
Convulsions from Worms . . 434
Cholera of India, remarks on . 73
Circulation, on the powers of, in an inflamed part . . . ib.
Contraction, muscular, on . 21
Cassowary, on the anatomy of . 165
Comparative anatomy and physiology of the Eye . . 13
Changes, organic, from inflammation 340
Catalogue of Books, 85, 173, 261, 348, 437, 525
Compression of Aneuiism, apparatus for . . . . 46

PAGE
Degeneration of Muscles . . 167
Destruction of Fœtal Brain, account of . . . 140
Diseases, feigned, on . . 87
——— of the Teeth, observations on . . . . . 189
——— resembling Cancer, on . 64
Distortions of the Spine, on the . 52
Digitalis, on large doses of . 476

Eye, observations on the physiology of . . . . . 7
——, on the anatomy of . . 3
——, comparative anatomy and physiology of . . . 13
Eritrogene . . . 259
Extraction of Calculi from the Bladder . . . 145
Epidemics, account of . 333, 417
Experiments on the Brain . 252
Exomphalos, strangulated, history of 149
Extirpation of Thyroid Gland . 42
——— the Parotid Gland, on the . . . . . 44
Experiment with Laudanum . 253
Errata . 86, 174, 262, 350, 438
Ear, on the minute anatomy of . 16
Earth-worm, on the reproduction of 23

Face, irritable Ulcers of . . 99
Fever and Dysentery, on . 149
——, remarks on . . 375
Functions of the Skin . . 218
Feigned Diseases, on . . 87
Fœtal Brain, destruction of the . 140
Fishes, peculiarities of the Brain in 2

Gymnastics, observations on . 199

Hæmorrhage, internal, case of . 289
Hydrocephalus Chronicus, case of 102
Hydrocyanic Acid, remarks on . 369
Hydrocele of the Spermatic Cord, on . . . . . 325
Hooping-cough, on the 247, 319 187

Inflammation of the Cellular Texture, cases of . . . 503
———, changes from . . 340
Injuries of the Thigh-bone and Spine 484
——— Shoulder-joint, remarks on . . . 49
——— Pelvis, on . . 146
Intermittents, Sulphate of Quinine in . . . . . 167
Inflamed part, powers of the circulation in . . . 73
Indians of Upper Canada, medicines used by the . . . 200
Iodine, observations on . . 311
———, on the use of . 341

PAGE
Inversion of the Uterus on . . 258
Infantile disease, cases of . . 498

Jalap, new alkali in . . 346

Ligature of the Subclavian Artery 147
——— Arteries, on the temporary . . . . . 46
Labour, complicated case of . 143
Laudanum, experiment with . 253
Lithotomy, observations on . 430
Lunatics, military, report on . 462

Muscular contraction on . . 21
Medical Report of the Hospital of Hobart Town . . . 519
——— Books, monthly catalogue of . 85, 173, 261, 348, 437, 525
Meconic Acid, remarks on . 344
Muscle, on the degeneration of . 167
Meningeal Apoplexy, case of . 364
Medicines used by the Indians . 200
Medicinal Saturation, on . . 283
M'Avoy's case, Miss, remarks on . 111
Meteorological Tables 86, 174, 262, 350, 438, 526
Military Lunatic Asylum, report of 462
Marum Verum, in Polypus of the Nose . . . . . 44

Nervous System, remarks on . 74
Neuralgia, cases of . . 271
Nicotiana, on the Tincture of . 256
Notices to Correspondents, 86, 262, 438

Observations on Rheumatism . 446
——— Scrofulous Diseases 410
——— Tartar Emetic, 276, 380
——— Gymnastics . 199
——— Iodine . . 311
——— the Sympathetic Nerve . . . 160, 232
——— Lithotomy . 430
Opening, fistulous, in the Trachea 254
Organic changes from Inflammation 340
Obituary . . . . 173
Operations Tagliacotian . . 42

Physiology of the Eye, on . . 7
Palate, ulceration of . . 389
Pneumo-thorax, remarks on . 32
Phthisis Pulmonalis, on the use of the Actæa Racemosa in . 256
Prussic Acid, remarks on . . 37
———, on a substitute for . 170
Phlegmasia Dolens, cases of . 129
Perforation of the Stomach . 433
Pathology of the Brain, on . 400
Pelvis, on Injuries of the . . 146
Parotid Gland, on the extirpation of 44

PAGE
Plates, references to, 175, 263, 351, 475
Pathology of the Spleen, on the . 439
Percussion and compression in Rheumatism, on . . . 446
Polypus of the Nose, on the Marum Verum in . . . 44

Quicksilver, effects of . . 171
Quinina, remarks on . . 138

Reproduction of the members of the aquatic Salamander . 22
——— in animals, on 22, 23
Ribs, Caries of the . . 114
Red Disease of Cayenne, account of 255
Remarks on Aneurism . . 30
——— Pneumo-thorax . 32
——— Stricture and Calculi 47
——— Retention of Urine . 168
——— Quinina . . 138
——— Meconic Acid . 344
——— Prussic Acid . . . 37
——— Spinal Diseases . 263
——— the Cholera of India . 73
——— Colchicum . . 181
——— the Teeth of the Guinea Pig . . . . 253
——— Hydrocyanic Acid . 369
——— the Brain . . . 1
References to Plates, 175, 263, 351, 475
Report, medical, of the Hospital at Hobart Town . . . 519
Royal College of Surgeons, new regulations of . . . 521
Rheumatism, observations on . 446
Reversed position of the Viscera, case of . . . . . 474
Rupture of the Liver, reference to cases of . . . . 37
Reference to cases of Acupunctuation . . . . 39

Stricture of Urethra, cured by incision . . . . . 142
Scrofulous Diseases, observations on 410
Spine and Thigh-bone, on injuries of 484
Skin, on the functions of . . 218
Stricture and Calculi, remarks on 47
Sulphate of Quinine in Intermittents 167
Shoulder-joint, remarks on injuries of . . . . . 49

PAGE
Strangulated Exomphalos . 149
Spinal Diseases, remarks on . 263
Stomach, perforation of the . 433
Small-pox after Vaccination, on, 68, 296
Sarsaparilla, on the virtues of . 72
Saturation, on medicinal . . 283
Sympathetic Nerve, observations on . . . . 160, 232
Substitute for Prussic Acid . 170
Spermatic Cord, on Hydrocele of 325
Spine, on distortions of the . 52
Stricture of the Urethra, remarks on . . . . 134
Surgeons, Royal College of, new regulations of . . . 521
Spleen, on the pathology of . 439
Salamander, aquatic, on the reproduction of the members of . 22

Tartar Emetic, observations on 276
380
Tumor on the Conjunctiva . 344
Thyroid Gland, extirpation of . 42
Twins, united, case of . . 292
Trachea, fistulous opening in . 254
Teeth, observations on the diseases of . . . . . . 189
——— of the Guinea-pig, remarks on . . . . 253
Tincture of Nicotiana on . . 256
Tables, Meteorological, 86, 174, 262, 350, 438, 526
Tagliacotian operation . . 42

Ulcers of the Face, use of Nitric Acid in . . . . . 99
Urethra, Stricture of the, cured by incision . . . . 142
——— on Strictures of the . 134
Ulceration of the Palate . . 389
Urine, retention of . . 168
Uterus, on the . . . 16
———, on the Inversion of . 258
United Twins, case of . . 292

Varioloid Disease, cases of . 299
Vaccination, on Small-pox after, 68, 296
Viscera, reversed position of . 474

Worms, Convulsions from, case of 434
Whooping-cough, on . . 247, 319

### INDEX.

### REVIEWS.

### DOMESTIC.

PAGE

On the Nature and Treatment of the Distortions to which the Spine and Bones of the Chest are subject; with an Inquiry into the Merits of the several Modes of Practice which have hitherto been followed in the Treatment of Distortions. By JOHN SHAW, Surgeon and Lecturer on Anatomy 52
Engravings in Folio, illustrative of the above Work.

Medico-Chirurgical Transactions, published by the Medical and Chirurgical Society of London. Vol. XII. Part II. . . . . . 64, 129

Practical Observations on Fever, Dysentery, and Liver Complaints, as they occur amongst the European Troops in India; illustrated by numerous Tables and Cases. To which is annexed, an Essay on Syphilis. By GEORGE BALLINGALL, M.D. F.R.S.E. &c. . . . . . 149

Lectures on the Anatomy and Functions of the Skin, delivered at the Royal College of Surgeons. By THOMAS CHEVALIER, Esq. F.R.S. Professor of Anatomy and Surgery to the College . . . . . 218

Pharmacopœia Collegii Regalis Medicorum Londinensis, M.DCCCXXIV.; and the same translated . . . . . . . 224

Essay on the Effects of Iodine on the Human Constitution; with practical Observations on its Use in the Cure of Bronchocele, Scrofula, and the Tuberculous Diseases of the Chest and Abdomen. By W. GAIRDNER, M.D. . . . . . . . . . . 311

Transactions of the Medico-Chirurgical Society of Edinburgh, instituted August 2, 1821; with Plates . . . . . . . 399, 498

Observations on Injuries of the Spine and of the Thigh-bone; in two Lectures delivered in the School of Great Windmill-street. By CHARLES BELL, Surgeon to the Middlesex Hospital . . . . . 484

### FOREIGN.

De l'Influence du Système Nerveux sur la Digestion, Stomach, &c. Par MM. BRESCHET, M.D.P. &c. H. MILNE EDWARDS, M.D.P. and —— VAVASSEUR, M.D.P.—(*Memoire la à la Societé Philomatique*) . . 74

De Nervi Sympathetici Humani, fabrica, usu, et morbis, Commentatio, Anatomico-Physiologico-Pathologica, &c. &c. Auctore J. F. LOBSTEIN 160, 232

An Essay concerning Tussis Convulsiva, or Whooping-Cough; with Observations on the Diseases of Children. By B. WATERHOUSE, M.D. late Professor of the Theory and Practice of Physic in the University of Cambridge (America) . . . . . . . 247, 319

Memoria sull' Idrocele del Cordone Spermatico. Di ANTHONIO SCARPA, &c. &c. . . . . . . . . . . 325

Leçons sur les Epidemics et l'Hygiene publique. Par F. E. FODERE. Tom. II. . . . . . . . . 333, 417

Recueil de Memoires, Consultations, et Rapports, sur divers Objets de Medicine Legale. Par M. CHAUSSIER, &c. &c. . . . 507

### MEDICAL AND SCIENTIFIC BIBLIOGRAPHY.

Practical Observations on the Removal of every Species of Cataract by Hyalonyxis, or Vitreous Operation, illustrated by Cases: with critical and general Remarks on the other Methods employed. By JOHN BOWEN, M.D. Member of the Royal College of Surgeons, London . . 423

PAGE

Symptomatology, or the Art of detecting Disease; a Lecture occasionally read to the Pupils of the Westminster Hospital, and published according to their request. By ALEXANDER P. BUCHAN, M.D. F.L.S. senior Physician to that Institution . . . . . . . . 427

An Essay on an improved Method of cutting for Urinary Calculi, or the Posterior Operation for Lithotomy. By W. W. SLEIGH, Member of the Royal College of Surgeons, and Lecturer on Anatomy and Surgery in London . . . . . . . . . . 430

### MISCELLANEOUS.

Inoculation with Syphilitic Matter . . . . . . . 260

Hunterian Society, Officers elected . . . . . 261

Medical Society, Officers and Council for the year . . . 347

Action to recover the Amount of an Apothecary's Bill . . . 436

Fothergillian Medal . . . . . . . . ib.

College of Surgeons, new Regulations of . . . . . 521

### OBITUARY.

Mr. GRAINGER . . . . . . . . . . 173

### MONTHLY CATALOGUE OF BOOKS.

Pages 85, 173, 261, 348, 437, 525.

### METEOROLOGICAL TABLES.

Pages 86, 174, 262, 350, 438, 526.

### NOTICES TO CORRESPONDENTS.

Pages 86, 262, 438, 526.

### ERRATA.

Pages 86, 174, 262, 350, 438.

### REFERENCES TO PLATES.

Mr. MORTON's Case of Malformation of an Infant . . . 175
Dr. HARRISON's Cases of Spinal Disease . . . . 263, 351
Mr. FOX's Case of Reversed Position of the Viscera . . . 475

### NAMES OF AUTHORS,

*Of whose Works, Observations, &c. either a detailed Account, or more or less general View, is given in*

THE FIFTY-FIRST VOLUME

OF THE LONDON MEDICAL AND PHYSICAL JOURNAL.

PAGE

Averill, Mr. on Irritable Ulcers of the Face . . . . 99
Anthony, Dr. case of Caries of the Ribs . . . . 114
Arnott, Mr. case of Stricture cured by Incision . . . 142
Allan, Mr. case of complicated Labour . . . . . 143
Andral, M. jun. on Aneurism of the Aorta opening in the Trachea . 341
Alison, Dr. on Scrofulous Diseases . . . . . 410

Bushell, Mr. case of Internal Hemorrhage . . . . 289

——— case of Cephalalgia . . . . . . 177

Balfour, Dr. on Emetic Tartar . . . . . . 276, 380

——— on Percussion and Compression in Rheumatism . . 446

Bell, Mr. C. on the Spine and Thigh-bone . . . . 484

——— on the Eye . . . . . . . . 7

Bowen, Dr. on Cataract . . . . . . . 423

Buchan, Dr. on Symptomatology . . . . . . 427

Balingall, Dr. on Fever and Dysentery . . . . 149

Breschet, M. on the Nervous System . . . . . 74

——— on imperfect Development of the Brain . . . 25

Brown, Mr. his case of Hydrocephalus Chronicus . . . 102

Brayne, Mr. cases of Biliary Calculi . . . . . 139

Burnett, Dr. on the Effects of Quicksilver . . . . 171

Bienvenu, M. case of Fistulous Opening into the Trachea . . 254

Bergeron, M. on the Red Disease . . . . . 255

Bezio, Sig. on Eritrogene . . . . . . 259

Benaber, Dr. case of Polypus Tumor . . . . . 344

Bacot, Mr. on Ulceration of the Soft Palate . . . . 389

Chevalier, Mr. on the Skin . . . . . . . 218

Civiale, M. on Stricture and Calculi . . . . . 47

Cumin, Dr. on Burns . . . . . . . . ib.

Cooper, Sir A. on extracting Calculi from the Bladder . . . 145

Cloquet, M. Jules, on Injecting the Bladder . . . . 169

Clias, M. on Gymnastics . . . . . . . 199

Calvert, Dr. on Fever . . . . . . . 375

Cormick, Mr. on the Cholera of India . . . . . 73

Chaussier, M. on Legal Medicine . . . . . 507

Davy, Dr. on Pneumo-thorax . . . . . . . 32

——— on large Doses of Digitalis . . . . . 476

Davis, Dr. D. on Phlegmasia Dolens . . . . . 129

De Rossi and Tonelli, on Sulphate of Quinine in Intermittents . 167

Dupasque, M. on Retention of Urine . . . . . 168

Duncan, Dr. jun. on Inflammation of the Cellular Texture . . 503

Desmoulins, M. on the Peculiarities of the Brain of Fishes . . 2

Elliottson, Dr. on Quinina . . . . . . . 138

Earle, Mr. on the Shoulder-joint . . . . . . 49

——— on Diseases resembling Cancer . . . . . 64

PAGE

FODERÉ, M. on Epidemics . . . . . . 333, 417

FLOURENS, M. Experiments on the Encephalon . . . . 252

FENOGLIO, Dr. on pure Meconic Acid . . . . . 344

FOX, Mr. his case of Reversed Position of the Viscera . . . 474

GAIRDNER, Dr. on Iodine . . . . . . . . 311

———— on Infantile Disease . . . . . 498

GIBBS, Dr. on Axillary Aneurism . . . . . . 147

GORE, Mr. case of Strangulated Exomphalos . . . . 149

GUERSENT, M. on the Degeneration of Muscles . . . . 167

GARDEN, Dr. on Actæa Racemosa in Phthisis Pulmonalis . . 256

GREGORY, Dr. G. on Small-pox after Vaccination . . . 68

HELLER, Dr. on Prussic Acid . . . . . . . 37

HEDENUS, Dr. on Extirpation of the Thyroid Gland . . . 42

HUTCHISON, Mr. COPLAND, on Feigned Diseases . . . 87

HAMMOND, Mr. on Destruction of the Fœtal Brain . . . 140

HARRISON, Dr. E. on Spinal Diseases . . . . 263, 351

HUTCHINSON, Mr. cases of Neuralgia . . . . . 271

HUME, Mr. jun. on a new Alkali in Jalap . . . . . 346

JOHNSON, Dr. J. case of Meningeal Apoplexy . . . . 364
JONES, Dr. on Small-pox after Vaccination . . . . 296
JUKES, Mr. Experiment with Laudanum . . . . . 253
JACOB, Dr. on the Anatomy of the Eye . . . . . 3

KOECKER, M. on the Teeth . . . . . . . 189

KNOX, Dr. on the Anatomy of the Cassowary . . . . 165

KINGLAKE, Dr. on Colchicum . . . . . . 181

———— on Medicinal Saturation . . . . . . 283

KERR, Mr. on the Medicines used by the Indians of Upper Canada . 200

KOLLEY, Dr. on the use of Iodine . . . . . . 341

KELLIE, Dr. on the Pathology of the Brain, and Death from Cold . 400

LOBSTEIN, M. on the Sympathetic Nerve . . . . 160, 232
LANGSTAFF, Mr. case of Ascites . . . . . . 144
LAENNEC, M. case of Perforation of the Stomach . . . . 433

MEDORO, Dr. on Temporary Ligature . . . . . . 46
MORTON, Dr. case of Malformation of an Infant . . . . 175
MAGENDIE, Mr. Injection of Warm Water into the Veins . . 255
MEPLAIN, Dr. case of Convulsions from Worms . . . . 434
MAYER, Mr. on Marum Verum in Polypus of the Nose . . . 44

PREVOST and DUMAS, MM. on Muscular Contraction . . . 21

———————— on the Blood . . . . . 18

———— on Decomposing Urinary Calculi . . . . . 40

PARIS, Dr. on Medical Jurisprudence . . 123, 206, 301, 392, 477

POPE, Mr. on Sarsaparilla . . . . . . . . 72

PHILIP, Dr. W. on the Powers of the Circulation in an Inflamed Part . 73

ROSTAN, M. on Softening of the Brain . . . . . . 28

RENWICK, Dr. on Miss M'Avoy's Case . . . . . 111

ROOTS, Dr. case of Bronchocele . . . . . . . 141

ROBIQUET and VILLERMÉ, MM. on a Substitute for Prussic Acid . 170

ROUSSEAU, Mr. on the Teeth of the Guinea-pig . . . . 253

RYAN, Dr. on Hydrocyanic Acid . . . . . . . 369

ROSENTHAL, M. on the minute Anatomy of the Ear . . . 16

SHARPLESS, Dr. case of Imperforate Anus . . . . 257
SCOTT, Mr. his Statistical Report . . . . . . . 519
SCHETKY, Mr. his Report on the Asylum for Military Lunatics . . 462

| | PAGE |
|---|---|
| San Giovanni, M. on the Reproduction of the Earth-worm . . . | 23 |
| Swan, Mr. on Injuries of the Pelvis . . . . . | 146 |
| Scarpa, Professor, on Hydrocele of the Spermatic Cord . . . | 325 |
| Sleigh, Mr. on Lithotomy . . . . . . . . . | 430 |
| Shaw, Mr. on Distortions of the Spine . . . . . | 52 |
| ——— on Stricture of the Urethra . . . . . | 134 |
| | |
| Tiedeman, Professor, on the Uterus . . . . . | 16 |
| ——— on the Brain . . . . . | 1 |
| Treviranus, Dr. on the Comparative Anatomy and Physiology of the Eye | 13 |
| Todd, Dr. T. T. on Reproduction in Animals . . . . | 22 |
| Teallier, Dr. on Inversion of the Uterus . . . . . | 258 |
| Toms, Mr. case of Male Twins united . . . . . | 292 |
| | |
| Villerme, M. Table of Organic Changes from Inflammation . . | 340 |
| Vetch, Dr. on the Pathology of the Spleen . . . . | 439 |
| Verdier, M. his Apparatus for Compression of Aneurism . . | 46 |
| | |
| Waterhouse, Dr. on Whooping-cough . . . . . | 247, 319 |
| Ward, Mr. on Aneurism . . . . . . . . | 30 |
| Weinhold, Dr. on Extirpation of the Parotid Gland . . . | 44 |
| Webster, Dr. on Hooping-cough . . . . . . | 187 |
| Westbury, Dr. on Tincture of Nicotiana . . . . . | 256 |
| Wolff, M. case of Imperforate Anus . . . . . | 258 |
| Wade, Mr. cases of Varioloid Eruption . . . . . | 299 |
| | |
| Yelloly, Dr. case of Diseased Arteries . . . . . | 148 |

### MONTHLY CATALOGUE OF MEDICAL BOOKS.

*** *It having been hinted to us that gentlemen, sending copies of their Works, prefer having their titles given at length, it is our intention, in future, to comply with this suggestion: but it is to be observed, that no books can be entered on this List except those sent to us for the purpose;—finding, in the lists hitherto transmitted, that the names of works have frequently been given as published, which have not appeared for weeks, or even months after.*

On the Nature and Treatment of the Distortions to which the Spine and the Bones of the Chest are subject; with an Inquiry into the Merits of the several Modes of Practice which have hitherto been followed in the Treatment of Distortions. Illustrated by Plates in Folio. By JOHN SHAW, Surgeon, and Lecturer on Anatomy.—pp. 293. London, 1823.

Observations illustrative of the History and Treatment of Chronic Debility, the prolific Source of Indigestion, Spasmodic Diseases, and various Nervous Affections. By W. SHEARMAN, M.D. Member of the Royal College of Physicians; President of the Medical Society of London; senior Physician to the Royal West-London Infirmary, and Physician to the London Dispensary.—London, 1824.

Observations on Colds, Fevers, Diseases of the Liver, and other Disorders; with Directions for treating them: chiefly intended for the use of Gentlemen residing at a Distance from Medical Advice, and for young Students. By DANIEL JOHNSON, formerly Surgeon in the Hon. East-India Company's Service.—pp. 87. London, 1823.

Hygiène Militaire, a l'Usage des Armées de Terre. Par le Chevalier J. RORN LOUIS DE KIRCKHOFF, Membre de la plupart des Academies et Societés Savantes de l'Europe; ancien Medécin des Armées et Hopitaux Militaire. Seconde Edition, revue et augmentée.—pp. 249. Anvers, 1823.

Practical Observations on Fever, Dysentery, and Liver Complaints, as they occur among the European Troops in India; illustrated by numerous Tables and Cases. To which is annexed, an Essay on Syphilis. By GEORGE BALLINGALL, M.D. F.R.S.E. Fellow of the Royal College of Surgeons of Edinburgh; Surgeon Extraordinary to the King for Scotland; Regius Professor of Military Surgery in the University of Edinburgh; and one of the Surgeons to the Royal Infirmary of that City. Second Edition.—pp. 305. Edinburgh, 1823.

Transactions, by the Medico-Chirurgical Society of Edinburgh.—Edinburgh, 1823.

### NOTICE TO CORRESPONDENTS.

*In addition to those formerly announced, Papers have been received from* Dr. Payne, Mr. Kerr, Mr. Yeatman, *and* an Army Medical Officer.

Dr. Von Buch's *polite letter has been received; it will afford us pleasure to comply with his wishes.*

